# The Hospital. Nursing Section

**Published:** 1905-01-28

**Authors:** 


### The Hospital.

### Nursing Section.

Contributions for this Section of "The Hospital" should be addressed to the Editor, "The Hospital," Nursing Section, 28 & 29 Southampton Street, Strand, London, W.C.

No. 957.—Vol. XXXVII. *SATURDAY, JANUARY* 28, 1905.

### Notes on News from the Nursing World.

### THE NEW QUEEN'S NURSES.

The most interesting personal feature of the official list of the new Queen's Nurses, whose appointment has been approved by Queen Alexandra, is the inclusion of Miss Katherine Twining's name. Miss Twining is the superintendent of the Maternity Charity and District Nurses' Home at Plaistow, whose excellent work is well known. Twenty-two of the nurses are to serve in England, a decrease of eight as compared with the figures last January; eight in Wales, an increase of one; seventeen in Scotland, a decrease of three; and thirteen in Ireland, an increase of one. Twenty-two of the nurses appointed in England received their district training in London, nine in Liverpool, four in Manchester, five in Birmingham, and four in Brighton; all but one appointed in Scotland in Edinburgh, and the whole of those appointed in Ireland in Dublin.

### PRESENTATION TO MISS BALFOUR.

The practical interest which Miss Balfour has for some years manifested in the nursing movement north of the Tweed has been acknowledged in a graceful manner on the occasion of the amalgamation of the Prestonkirk Benefit Association with the East Lothian Association. The members of the former organisation have presented her with an address, which expresses to Miss Balfour, as the hon. treasurer, the Society's heartfelt appreciation of the benefits which she has conferred on the respective parishes, and on the county of East Lothian at large, by the full development of her scheme. It is noteworthy that though the Prime Minister's sister has so many claims upon her attention, she should be able to find time to render valuable and continuous assistance to the work of nursing the sick poor in Scotland.

### THE DELAYED NURSING ORDER.

At a meeting of the Council of the Poor-law Unions' Association of England and Wales last week, the question of the issue of the long-deferred nursing order was raised. The secretary reported that he had only received a formal acknowledgment from the Local Government Board to the request that they would promulgate the new nursing order without further delay. Dr. Rhodes and other speakers said it was time that the order was issued, and we agree with them. Unless it makes its appearance at a very early date, people will naturally be confirmed in the impression that Mr. Walter Long intends to regard the exhaustive report of the Departmental Committee as so much waste paper. While we strenuously dissent from their proposal to create a person called a qualified nurse, her qualification being quite inadequate training, there are many admirable recommendations in the report which we hope that the Local Government Board will adopt.

### THE PASSING OF THE INCOMPETENT MIDWIFE.

At an inquest in Battersea last week on the body of the wife of a labourer the midwife who attended the woman stated that she was not registered, but that a doctor had "learned her all he could." She was paid 7s. 6d. for attending each case, the attendance lasting ten days. Asked by the coroner whether she used a clinical thermometer the midwife replied, "I don't understand what you say." She proceeded to mention that she had acted as a midwife for 32 years, and at the present time was attending eleven cases. Medical evidence was to the effect that the cause of death was sudden failure of the heart, but the opinion was expressed that a competent midwife would have discovered, by the rise of temperature, that there was something wrong. The coroner, in summing up, observed that the case belonged to the class which would be dealt with by the Midwives Act. He described the midwife as a well-meaning woman who had undertaken work for which she was not qualified, and he warned her that if she undertook other cases "he was perfectly certain that a jury would not again overlook her incompetence if any serious consequences followed." This the jury evidently interpreted as a suggestion that they should only attribute accidental neglect, and no doubt they were right. But the urgent need for the Act which will prevent deaths from the unintentional neglect of incompetent midwives has once more been sadly illustrated.

### THE IRISH BENEVOLENT FUND.

In spite of a good deal of pushing, the organisation called "King Edward the Seventh's Coronation National Fund for Nurses" does not seem to make much progress. At the last meeting of the council in Dublin a few days ago, the problem of how to increase the receipts was anxiously considered. With that object in view, circulars are being issued to the general public, calling their attention to the fact that "this benevolent fund has been established for the benefit of certificated and probationer nurses in Ireland, who through ill-health, adversity, or old age, have become incapacitated for work." It will not be surprising if the Irish public continue to refrain from assisting a distress fund in the interest of "probationer nurses" who are, or should be, young women able to earn a living.

### AN IMPROVEMENT AT STAFFORD UNION INFIRMARY.

Although, as a Poor-law Nurse says in her letter to us to-day, the Northallerton Guardians are not the only board who think that a nurse ought to be able to undertake unreasonable obligations, there are many boards who are more considerate. The Stafford Guardians, acting upon a suggestion from the Local Government Board, have just abandoned the practice of keeping one nurse permanently on night duty. Henceforth it is to be divided. The nurse whose turn it is to be on night duty is also to have the assistance of a probationer. The arrangement is in accordance with the wishes of the superintendent nurse, and it is a course which has been rendered possible by the appointment of two probationers in place of the former night nurse. We hope that this mode of dealing with night nursing will commend itself to guardians of other small infirmaries who are anxious that patients shall have adequate attention, and that willing nurses shall not be overworked.

### A DEFICIENCY AT PERTH.

EXCEPT for the announcement of the honorary treasurer, the proceedings at the annual meeting of the Perth Sick Poor Nursing Society were very satisfactory. The slight excess of expenditure over income would be more serious if the society did not possess a credit balance of nearly £1,000 at the bank. But this is capital, and we agree with the various speakers that the moderate amount of £292, which represents the cost of carrying on the work of the organisation for 12 months, ought to have been covered by contributions. The society has certainly made remarkable progress since it was founded 20 years ago. The nurses last year paid upwards of 7,500 visits, and have become a recognised power for good in the town. It is curious that the falling off is in the amount received by the lady collectors, which has hitherto been the largest source of income. We anticipate that now they are put upon their mettle, they will take care that the ground which has been lost is regained by the time the next report is issued.

### THE REJOINDER OF THE SUPERINTENDENT NURSE AT PLYMOUTH.

THE superintendent nurse of Plymouth Workhouse Hospital has sent the Guardians a reply in which she entirely denies that her arbitrary conduct has been the cause of the Guardians being unable to retain the services of certain nurses. She says that the reasons for some resignations were unconnected with her, and that, as to the others, most of the nurses who resigned were far from being highly efficient. She mentions that several of the nurses who have resigned write to her periodically and come to see her when they are in Plymouth, and she urges that they would not do this if they left through any high-handed treatment on her part. Miss Halliday, turning the tables upon the Guardians, states that when appointed, over four years ago, she found the infirmary in an unsatisfactory condition as regards discipline and the care of the sick, and, with a view to remedying this state of affairs, asked the Guardians on no fewer than four occasions to draw up rules for the guidance of the nurses in the performance of their duties, but up to the present time she has not been furnished with any. She cites figures to indicate that it would be difficult to find another infirmary which will bear comparison to Plymouth so far as length of service is concerned, and she reminds the Guardians that on her election as superintendent nurse she was informed that the staff had been incomplete for some time, and that on taking up her duties she found that there was not a single assistant nurse who had completed one year's service. It seems to us that if the matter be referred to the Local Government Board the superintendent nurse will be able to give a good account of herself.

### NURSING ASSOCIATIONS AND BRITISH WORKMEN.

AT the annual meeting of the Darlington Queen's Nurses' Association, the Mayor, who moved the adoption of the report, expressed his regret that the balance against the treasurer had increased from £17 to £31, and having noted that the workmen belonging to one firm in the town had contributed to the fund, said he hoped that their example would be followed by other workmen. Bearing in mind the number of workmen employed at Darlington, a personal canvass among them, carried on not intermittently, but at regular times would probably do much to bring about the result desired by the Mayor. We believe that there are many towns in the United Kingdom in which bodies of British workmen could render small but regular assistance to district nursing associations if they were persistently and tactfully invited to con tribute. Of course, when they give in this way, care should be taken not to ask them again in their own homes.

### FAILURE OF ANOTHER NURSING HOME.

PRIVATE nurses possessing a little capital of their own which they cannot afford to speculate with, should be careful not to yield to any temptations to start a nursing home. Neither is it any better to commence on borrowed capital. These homes are already far too numerous, and the result is frequent financial disaster. At the Poole Bankruptcy Court the other day the proprietress of an establishment at Bournemouth came up for public examination, her liabilities being £1,053 against assets £163. The bankrupt stated that she had made three attempts to maintain herself in this way, and that the greater portion of her liabilities consisted of loans payable to people who desired to assist her. She attributed her failure to the increase of competition, and we have no doubt that this was, at any rate, one cause of it. The registration of nursing homes, by rendering it more difficult to open them, would prevent a good deal of money from being thrown away.

### MATERNITY TRAINING AT BELFAST.

AN important recommendation with reference to the new nursing scheme concerning the training in Belfast Union Maternity Hospital has just been adopted by the guardians. As it is only possible for sixteen nurses to receive the maternity training in the course of each year they are, in future, to be the sixteen who obtain the highest number of marks at the examination held at the end of their two years' general training. In the event of any of these not desiring to enter the Maternity Hospital the next nurse on the examination list will be afforded the opportunity. All nurses who take the maternity training will be required to enter for the midwifery examinations. This seems a very fair way of dealing with the matter.

### HOUSE-TO-HOUSE COLLECTIONS.

The committee of the Swansea District Nursing Association have marked the opening of the new year by starting a house-to-house collection. Ten ladies offered their services, and each of them has under her direction a board of collectors. We do not doubt that the result of the new departure will be a considerable increase in the receipts. Persistent house-to-house collections, carried on with tact, are the best means of obtaining funds for a nursing organisation. If the work is done thoroughly in Swansea, it will, at any rate, be impossible for anyone to plead ignorance of the objects of the Association, and for the few persons who may possibly resent the visit of the collector, there will probably be many who will give a practical response.

### QUEEN ALEXANDRA'S NAVAL NURSING SERVICE.

Five additions have been made to Queen Alexandra's Royal Naval Nursing Service. The new sisters are Miss E. M. Seeley, Miss A. E. Hartley-Jones, Miss L. A. Lowden, Miss N. G. M. Nigel Jones, and Miss F. M. Leale.

### MISS McCORD'S NEW APPOINTMENT.

We understand that Miss McCord, late matron of Queen Charlotte's Hospital, has left London in order to take up her new appointment, which is in the Italian Royal household. Miss McCord, at the personal invitation of Queen Elena, has gone to Italy to take entire charge of the Royal nurseries and devote her special attention to the up-bringing of the infant Heir to the Throne.

### NURSING THE VICTIMS OF THE CUDWORTH RAILWAY ACCIDENT.

The railway accident at Cudworth on the Midland line took place between 3.30 and 4 o'clock on Thursday last week. Somewhere about 7.30 on the same morning the first of the nine sufferers brought into Sheffield—distant about 18 miles—reached the Royal Hospital in one of the Sheffield City ambulances. At 8 30 the last of the nine, all males, had been brought to the hospital. In the hospital they were received in the casualty room, where the surgeons were in readiness for them. Two, apparently only slightly injured, returned to their homes in Sheffield after receiving immediate treatment. The remainder were distributed in five different wards, two being placed in small wards and the others in the general surgical wards. In the course of the morning three of the seven were anæsthetised, underwent operative treatment, and returned to the wards under charge of special nurses. In one of these operation cases the after treatment was complicated by the presence of extensive burns, and unfortunately this patient succumbed to his injuries on the third day after admission. The six patients now in hospital are progressing favourably, but are all still in need of special attention. There is no staff of private nurses attached to the hospital, and, owing to the fact that the hospital staff was already dealing with an unusual number of patients, many of them serious cases, a quite exceptional amount of work has been required from the nurses. This, however, has been most willingly undertaken, and has been made pleasant by the universally cheerful demeanour and plucky conduct of the patients. Although in each case the injuries received were very considerable, and the shocking scenes of the accident were recalled on the first night after admission by rambling fears of attacking steam-engines, in each the subsequent pain and discomfort, and in one case the approach of death, have been met with gallant stoicism.

### THE CENTRAL MIDWIVES BOARD AND THE LONDON OBSTETRICAL SOCIETY.

The Steyning Guardians have been informed by the Central Midwives Board that up to March 31 any holder of the certificate of the London Obstetrical Society is entitled, on payment of a fee of 10s., to receive the certificate of the Board; but that after that date all applicants, of whatever description, for the certificate of the Board will be required to pass the Board's examination. This is obviously an intelligible position, and it places all candidates on the same footing. That is to say, the question after the Midwives Act comes into force on April 1st will simply be whether she can pass the examination of the Board.

### QUEEN'S NURSES AT READING.

The annual report of the Queen Victoria Nursing Institute is not altogether satisfactory from a financial point of view. The balance in the hands of the bankers at the end of the previous year is £71 16s., a decrease of £15 11s. 6d., and the donations also show a decrease of £34 9s. 8d. On the other hand, the subscriptions, which should be the backbone of a nursing organisation, have increased to the extent of £20 15s. 2d. The collections in the churches were again less, but the members of the Ladies' Auxiliary must be congratulated on the fact that they collected upwards of £140. The record of work done is highly creditable. The number of patients nursed was 437, as against 425 in 1903, and the number of visits paid was 12,474, as against 13,165. But the cases as a whole were more serious, involving visits of longer duration. It is satisfactory to learn that the committee have shown their gratification at the manner in which the lady superintendent and the nurses have discharged their duties by giving an increase of salary to them, and it is proposed to appoint an additional staff. With Mr. Martin J. Sutton as president in his capacity of Mayor of Reading, the present year should be a thriving one for the Nursing Institute.

### DISTRICT NURSING AND DANCING.

There was a large attendance at the annual dance in aid of the Cardiff branch of Queen Victoria's Jubilee Institute. More elaborate arrangements were made than on any previous occasion, and the purchase of tickets was strenuously urged upon the public. The most striking argument in favour of supporting the Queen's nurses in Cardiff is supplied by the fact that last year they paid 62,334 visits and dealt with 1,858 cases, an increase in visits of 7,753 over 1903, and in cases of 278. This, of course, means that, to meet the needs of the sick poor in the town, the staff must be augmented. But Cardiff, with all its enterprise in municipal matters, is not likely to be irresponsive to the call.

### The Nursing Outlook.

"From magnanimity, all fear above;
From nobler recompense, above applause,
Which owes to man's short outlook all its charm."

### THE EMIGRATION OF POOR-LAW CHILDREN.

THE result of sending children to Canada has been so satisfactory that we are willing to welcome any proposed extension of it. Hitherto it has been most largely undertaken by voluntary agencies, such as Dr. Barnardo's, while boards of guardians, which have thousands of children in their care, have seemed to lag behind. This point is dwelt upon by Mrs. Elinor Close, who has lately brought out an attractive and in many respects practical scheme of child emigration. She desires guardians to lease farms of two or three hundred acres in some part of the Canadian Dominion, which would form the home of from fifteen to twenty children, under the care of two ladies, as many servants, and a practical farmer. Children would be taken from two years old and upwards, would attend the ordinary elementary schools of the neighbourhood where they lived, and in their spare time would learn kitchen, stable and farm work. It is proposed that when the children reached the age of 12 or 14, before they are withdrawn from the charge of the guardians, such as could be satisfactorily placed in situations in England should be returned there. The plan is, in short, an extension oversea of the scattered home system which some boards have adopted at home.

We think so highly of Mrs. Close's scheme that it is in no carping spirit that we venture upon some criticisms of it. First, she seems to think that guardians are averse from emigrating the children under their care. That is not the case, but the power which the guardians have over such children is strictly limited. The children sent abroad by voluntary agencies belong to these agencies absolutely. They are orphaned, homeless, or deserted. There is no one to interfere with Dr. Barnardo, for instance, when he sends a batch of boys and girls to Canada. But, while some such children come under the poor-law, a great many pauper children have at least one parent living. The barrack schools, whose cost and management Mrs. Close very reasonably condemns, are to a large extent orphanages. With some boards it is a popular way of helping a widow to take some of her children into the schools. The children may remain there for years, but the mother's claim to them does not cease, and it would be impossible to send them abroad without her consent.

It is safe to say that in very few cases would this consent be given. Natural affection added to the anxiety that comes from ignorance would prompt the refusal. Only those who have to do with the recipients of State aid know how difficult it is to move them from their accustomed environment whatever advantages are offered. Not long since a Metropolitan board had to cancel the apprenticeship of a boy to a good business in Leicestershire because the lad's mother objected to his going "so far away." An example of the difficulty which guardians find in dealing with children may be given, though it has nothing to do with emigration. The schools belonging to one metropolitan union being overcrowded, the guardians wished to board some with another union which had accommodation available. The latter, however, refused to take "infants," *i.e.* children under five, and "in and out" children. So difficult was it found to comply with these two simple-looking conditions that of 140 children only eight could be sent away. This is only one example of the difficulty of placing children outside the bounds of the schools of their own union.

The only children whom guardians could definitely undertake to emigrate would be those whom they have adopted, who number about 7,000. That a number of possible emigrants might be found among these is likely, but it must be remembered that these adopted children include a great many who by reason of the hardships and cruelty they have suffered from, might very reasonably be rejected by the Colonial Government. That Mrs. Close intends to include among her young emigrants children who may be defined as sub-normal, if not abnormal, we infer from the letter she has received from Sir James Crichton Browne. Sir James specifies as children who would benefit by such a scheme, "children unsuited for boarding out, and requiring special supervision—sickly children *not* actually diseased, difficult children *not* actually criminal, backward children *not* actually weak-minded, who would not be passed by the existing agencies." But the limitations which hamper existing agencies are set by the government of the country to which they send the children, and would equally hamper Mrs. Close.

For such children as could be sent the plan would be undoubtedly advantageous, and Mrs. Close's idea of taking quite young children—two or three years old—is good. Many children who, born healthy, become delicate in insanitary surroundings, would grow up strong and healthy in a purer air, and leading a country life. Whether it would be wise to return children brought up in the freedom of a Canadian farm to the more limited environment which would be open to them in the old country is a question which admits of difference of opinion. If suitable situations offered themselves in the adopted country, it would be unfair to the children not to let them accept these. But such matters usually settle themselves. While we see difficulties in the way of carrying out Mrs. Close's scheme on a large scale, we are sure that it would form a practical and valuable addition to the existing agencies for dealing with the children of the State.

### Lectures upon the Nursing of Infectious Diseases.

By F. J. WOOLLACOTT, M.A., M.D., B.S.Oxon, D.P.H., Senior Assistant Medical Officer, Park Hospital, Metropolitan Asylums Board, Hither Green.

### LECTURE XIII.—THE COMPLICATION OF MEASLES. GERMAN MEASLES.

THE complications of measles are important and constitute the chief danger of the disease. The most common are affections of the respiratory passages and the lungs. Probably a certain amount of bronchitis is usually present in the acute stage, but not infrequently the inflammation extends deeper into the lungs and sets up some form of *pneumonia.* As a rule this happens while the rash is still out or while it is fading, but occasionally during convalescence. The symptoms are much the same as in pneumonia due to other causes, but there is always considerable danger, as the patient may be much weakened by his previous sufferings. The temperature does not fall at the usual time, or, if it has fallen, it rises again. The cough is intensified, the breathing becomes rapid and often painful, and there may be some cyanosis. In many cases the progress is slow, and the continued pyrexia may cause much exhaustion and emaciation. As a rule there is more or less pleurisy, and if fluid is poured out into the pleural cavity it usually becomes purulent and gives rise to an empyema.

In every case of measles the possibility of these lung complications should be borne in mind from the commencement, and the safeguards described in the last lecture should be adopted, for by keeping the patient in a warm, even temperature and carefully protected from draughts and chills we may hope to carry him through in safety. When pneumonia has actually set in, the same general treatment should be continued, and, in addition, a cotton-wool jacket should be worn to protect and keep warm the chest. If a poultice be ordered, it should be applied to the back only, for, if wrapped round the chest, it may hamper the respiratory movements. The patient's strength must be maintained by food given at intervals of about two hours, and stimulants are often useful. If an empyema forms, an operation is necessary to let out the pus and drain the pleural cavity.

Another complication involving the respiratory passages is *Croup*, the result of inflammation of the larynx. Almost invariably it begins early in the disease, before the rash has appeared, or while it is coming out. The symptoms are similar to, but on the whole milder than those described in the lecture on diphtheritic croup. In some cases tracheotomy or intubation may be necessary, but very frequently the use of the steam kettle and the other measures previously mentioned will effect a cure. Relief, too, may be afforded by the application of hot compresses over the larynx, or of a leech to the base of the neck over the top of the sternum. Occasionally croup makes its appearance during the convalescent stage, and is then nearly always of a true diphtheritic character, and attended with the formation of membrane. Before the introduction of antitoxin this combination of measles and diphtheria was very frequently fatal, but now a prompt application of the remedy will usually be successful.

A second group of complications includes various forms of inflammation, which result from aggravation of the catarrhal symptoms present in the early stage of measles, and chiefly affect the eyes, mouth, and pharynx.

They are comparatively rare in well-to-do patients, who receive proper attention, but are common in weakly, neglected children, living in insanitary surroundings. Thus the early conjunctivitis may extend and cause severe inflammation of the eyelids, or ulceration of the cornea, and perhaps lead to chronic ophthalmia and imperfect sight. These conditions are difficult to cure after having once begun, but may usually be prevented if the eyes have from the start been carefully cleansed and protected from irritation.

As we have already seen, the parts inside the mouth are apt to become sore, and should be carefully attended to, otherwise troublesome ulceration may follow. The teeth especially should be regularly cleaned. In rare instances, and practically only in ill-nourished children, the condition may go on to a form of gangrene known as *cancrum oris.* The first sign is usually a red swelling of the cheek or lip, corresponding to a foul ulcer inside the mouth. The swelling quickly increases, and the part turns black and sloughs away. The process is very rapid, and can only be checked by prompt and vigorous treatment; so that the nurse should never fail to report as soon as possible any unusual condition of the mouth. Similar forms of ulceration and gangrene may attack the vulva and need equally prompt attention. Any swelling or discharge should be reported. Unfortunately these cases usually end fatally, and if recovery takes place, great deformity is left as a result of the loss of tissue and consequent scarring.

A more common complication belonging to this group is the occurence of *Ear discharge*, due to the extension of inflammation from the throat along the Eustachian tube to the ear. The process is the same as in scarlet fever, and the appropriate treatment was described in the lecture on the complications of that disease.

Another, and at times very formidable complication, is *Diarrhœa*, sometimes accompanied by vomiting, which usually begins during the period of the rash, or even earlier. If profuse it may cause extreme prostration, especially in young children. Apart from the use of drugs and stimulants, it is treated by careful attention to the diet, and peptonised milk, raw meat juice, and albumen water may all prove of service. Care must be taken to keep the patient warm by means of blankets and hot bottles, and benefit may result from the warm bath.

After an attack of measles complete recovery usually follows, although at times the patient is left delicate and weakly, and does not regain his normal health for some months. In these cases a change of air to the country or seaside is invaluable. The child should not be allowed to go out in inclement weather, should be warmly clothed and well fed. In other cases more or less permanent ill effects may be left. As we have seen defective vision may result from ulceration of the cornea, and deafness may follow inflammation of the ear. Pneumonia also occasionally leaves the lungs permanently weakened, and may even prove the starting point of phthisis. It is a curious fact, too, that during and immediately after measles there is a strong susceptibility to the infection of whooping cough, and it is, indeed, not uncommon to see the two diseases in progress at the same time.

A case of measles, if uncomplicated, continues to give off infection for about three weeks from the appearance of the rash, and if complications, especially pneumonia, be present, for a still longer time. Isolation, as strict as possible, should be enforced during the whole of the time.

The infection is probably most intense in the acute stage, during the catarrhal symptoms, the chief sources of danger being the discharges from the eyes, nose and mouth, together with the sputum. These discharges should be removed by pieces of rag or lint and immediately burnt. As a rule the nurse runs but little risk, for, as few people escape the disease in childhood, she has probably already had it, and second attacks are rare.

*German Measles, or Rubella.*—German measles, or rubella, though often confused with measles as the symptoms are somewhat similar, is a quite distinct disease. It is important to distinguish carefully between the two, as they differ very markedly in severity, the former being almost invariably of a mild character and without danger to life, while, as we have seen, the latter is frequently fatal. In the following description the chief differences will be mentioned. As a rule German measles shows a tendency to attack adults and older children rather than those in the first years of life. It is decidedly infectious, and is usually transmitted directly from person to person. The incubation period is a long one, and somewhat variable, 18 days being the average. Very frequently the patient has no feeling of illness whatever, and the disease is only recognised by the appearance of the rash. Occasionally, however, there is slight malaise for a day or two, with mild catarrhal symptoms, such as cough, sneezing, watering or smarting of the eyes, and slight sore throat. The glands at the back of the neck and behind the angle of the jaw are nearly always enlarged from the commencement, and sometimes tender, but they do not become acutely inflamed and never suppurate. The temperature may be raised a few degrees, or may be normal throughout.

The rash appears early, within a day or two of the first symptoms. It consists of pink or rose-red papules, which increase in size and coalesce to form slightly raised blotches. It begins on the face, usually on the parts round the mouth and nose, and soon spreads to the trunk and the rest of the body. As a rule it is not so thick as the rash of measles, and of a brighter colour. In practically every case the disease is of short duration. The rash fades, beginning on the face, the temperature falls, and any symptoms which may be present soon disappear. There is no staining of the skin left, though occasionally a fine powdery desquamation may follow, especially on the face. A curious and important peculiarity of the rash remains to be mentioned. In some cases it loses its blotchy character after a few hours or a day, and becomes diffused, and in places punctate, so that its appearance then closely resembles that of scarlet fever. So marked is the similarity that there may be the greatest difficulty in distinguishing between the two diseases. Complications of any sort are almost unknown, and recovery is complete in every way, no ill consequences being known to follow.

The treatment is very simple. The patient should be confined to his room, and if the temperature be raised, should be kept in bed, on a simple diet, until it falls. Isolation should be enforced during the attack and for a few days afterwards. The danger of infection is usually over in about 10 days, but may persist as long as three weeks. Although German measles is of so mild a character, it must not be forgotten that similar precautions should, as far as possible, be observed as in the other more serious infectious diseases. After recovery, the patient should be bathed before being allowed to associate with other people, and the clothes and bedding should be disinfected.

### Where to Go.

THE London Glove Company's Sale, Monday, January 30th, at 45 and 45A Cheapside, and 82 and 83 New Bond Street, W.

### Queen Victoria's Jubilee Institute for Nurses.

HER MAJESTY QUEEN ALEXANDRA has been graciously pleased to approve the appointment of the following as Queen's Nurses, to date January 1st, 1905:—

### ENGLAND.

| Nurse | District Training at | Working at |
|---|---|---|
| Katherine Twining | | (Superintendent) Maternity, Charity, and District Nurses' Home, Plaistow |
| Annie Napier Andrew | Bermondsey | Bermondsey |
| Amelia Strachan | " | Darlington |
| Elizabeth Ellen Breakspear | Birmingham (Moseley Road) | Birmingham |
| Ethel Cole | " | " |
| Anne Dutton | " | " |
| Charlotte Ann Maskell | Birmingham (Newhall Street) | " |
| Annie Oulsnam | " | " |
| Isabel Beseke | Bloomsbury | Kettering |
| Marietta Alexandra Frances Carnochan | " | Bedford |
| Mary Grant McDougall | " | Gainford |
| Katharine Mackay | " | Trumpington |
| Alice Agnes Pedler | " | Cookham |
| Muriel Wyndham van der Meulen | " | Hertford |
| Agnes Leneghan | Bolton | Bolton |
| Lilian Friend | Brighton | Warsop |
| Annie Elizabeth Jones | " | Brighton |
| Margaret Louisa Kingston | " | St. Helens |
| Ethel Elizabeth Taylor | " | |
| Susannah Harriet Taylor | Camberwell | Camberwell |
| Alice Dale | Cardiff | Pontypool |
| Annie Houghton | " | Carlisle |
| Mary Rihoy | " | Kimpton |
| Elizabeth Margaret Roberts | " | Manchester (Ardwick Green Home) |
| Mary Kennedy Wylie | Carlisle | Carlisle |
| Jessie Wilhelmina Richmond | Chatham | Chatham |
| Marie Caroline Turpin | " | " |
| Beatrice Louisa Ford | Coventry | Coventry |
| Louie Taylor | " | " |
| Mary Ellen Edwards | Didsbury | Didsbury |
| Catherine Eleanor Finnis | East London (Central Division) | East London |
| Florence Mary Moles | " | " |
| Eveline Higgs | East London (Southern Division) | Paddington |
| Janet Mabel Westwood | East London (Stepney Green Division) | East London |
| Eva Frances Knight | Gloucester | Lowther |
| Catherine Elinor Crowther | Hammersmith | Hammersmith |
| Jessie Scott Robson | " | " |
| Sophy Anne Perrott Field | Hayle | Hayle |
| Amy Bounds | Horsforth | Horsforth |
| Elizabeth Alice Pearce | Kingston-on-Thames | Kingston-on-Thames |
| Agnes Gardiner | Central Home, Liverpool | Liverpool |
| Elizabeth Grenfell | Central Home, Liverpool | " |
| Annie Elizabeth Taylor | Central Home, Liverpool | " |
| Frances Gibson | Derby Lane Home, Liverpool | " |
| Jessie Burgess | Kirkdale Road Home, Liverpool | " |
| Selina Roberts | Overton Street Home, Liverpool | Hayes |
| Martha Borthwick Ewart Munro | Shaw Street, Home, Liverpool | Liverpool |
| Catherine McFarlane Peter | " | " |
| Annie Silversides | " | Doncaster |
| Helen Pitman | Ardwick Green Home, Manchester | Staveley Town |
| Susan Morrison | Bradford Home, Manchester | Manchester |
| Amy Bradley | Hulme Home, Manchester | " |
| Martha Foster | " | " |
| Amelia Sophia Gibson | Portsmouth | Manchester (Bradford Home) |
| Edith Isabelle Lloyd | " | Stafford |
| Lucy Parker | Salford | Huddersfield |
| Florence Pickthall | " | Salford |
| Ada Annie Walthew | " | Warrington |
| Mary Harriet Bevington | Sheffield | Sheffield |
| Janet Bray | " | " |
| Ellen Laura Wells | " | " |
| Clara Woodhead | " | " |

| Nurse | District Training at | Working at |
|---|---|---|
| Louisa Ball | Shoreditch | Shoreditch |
| Katie Emma Bangham | ,, | ,, |
| Margaret Hunter | ,, | ,, |
| Mary Ann Logan | ,, | Woolton |
| Constance Mary Percival | ,, | Paddington |
| Frances Laura Annie Williams | Southampton | Kettering |
| Elizabeth Pepper | Sunderland | Sunderland |
| Mary Jane MacIvor | Taunton | Taunton |
| Gertrude Maud Hammond | Walworth | Bedford |
| Jennie Younger | ,, | Rawtenstall |
| | WALES. | |
| Susan Alice Perkins | Bangor | Bangor |
| Elizabeth Ellen Jones | Bermondsey | Neath |
| Mary FitzGerald | Cardiff | Cardiff |
| Winifred Ethel Nicholas | ,, | ,, |
| Alice Maud Matthews | Paddington | |
| Frances Emily Bridges | Portsmouth | Cardiff |
| Sarah Ann Pryce | Shoreditch | Ammanford |
| Ellen Williams | ,, | Llanfaethlu |
| | SCOTLAND. | |
| Robina Anderson | Edinburgh | Edinburgh |
| Jeannie Ronald Carnie | ,, | ,, |
| Ella Margaret Carr | ,, | ,, |
| Helen Elizabeth Denholm | ,, | ,, |
| Jessie Dolina Douglas | ,, | Fair Isle |
| Barbara Gibson Hannah | ,, | Glendaruel |
| Isabella Maccallum | ,, | Ardrishaig |
| Jessie McIntosh | ,, | Aberdeen |
| Sophia Barnett Mackinnon | ,, | Connel |
| Grace Pearson Montgomery | ,, | Edinburgh |
| Mary Rennie | ,, | Jedburgh |
| Helen Clark Runciman | ,, | Edinburgh |
| Agnes Isabella Sutherland | ,, | ,, |
| Mary Thomson Stewart | ,, | Stornoway |
| Helen Thomson | ,, | Edinburgh |
| Annie Mackenzie Watt | ,, | Paisley |
| Sarah Ellis | Aberdeen | Aberdeen |
| | IRELAND. | |
| Honora Mary Barron | St. Lawrence's Home, Dublin | Dundalk |
| Ellen Ann Donald | ,, | Inverin |
| Rosina Hannigan | ,, | Dublin |
| Letty Lynch | ,, | Cappoquin |
| Eileen Norah Moloney | ,, | Newmarket-on-Fergus |
| Mary Kathleen O'Neill | ,, | |
| Susan Wedman | ,, | Dublin |
| Margaret Irvine | St. Patrick's Home, Dublin | Armagh |
| Mary Elizabeth Laughlin | ,, | Londonderry |
| Margaret Sarah Middleton | ,, | Dublin |
| Ida Peile | ,, | ,, |
| North Elizabeth Smyth | ,, | ,, |
| Elizabeth Watson | ,, | Limavady. |

### Central Midwives Board.

A SPECIAL meeting of the Central Midwives Board, summoned under the rules of procedure on the proposed removal of a name from the Roll, was held at the board-room on Thursday, last week. There were present Dr. Champneys (chairman), Dr. Ward Cousins, Dr. Cullingworth, Mr. Fordham, Mrs. Latter, Miss R. Paget, Miss Wilson, and Mr. Parker Young.

### THE CASE OF MISS EDITH GREGORY.

The business before the Board was the hearing of charges alleged against Miss Edith Gregory, a certified midwife, of signing false certificates in connection with the training of candidates for the examinations of the London Obstetrical Society. Miss Gregory was present with her solicitor.

The circumstances, as appeared from the correspondence read by the Secretary of the Board, were briefly as follows. The Obstetrical Society of London, having become aware in June of last year that the schedules presented before examination by certain of Miss Gregory's pupils, had been signed by her before the candidates had actually completed the 20 cases vouched for as having been attended by them "prior to the date of signature," to her satisfaction, instituted an inquiry, and in the result decided at a meeting of the Council in August 1904 to remove Miss Gregory's name from their books and to cancel her certificate. These facts were brought to the notice of the Central Midwives Board, and Miss Gregory was summoned before them to show cause why her name should not also be removed from the Midwives Roll, upon which she was admitted by virtue of the certificate of the Obstetrical Society of London

Miss Gregory, in her defence, admitted the signing of the schedules, stating that she had been in the habit of so doing before the completion of the cases, when pupils were satisfactory, and the cases booked were sufficient to make up the required number before the actual date of the examination. Pupils could always remain with her until they had completed their full number. She did this because the schedules had to be sent in a month before the examination, and with no intent improperly to secure admission for her pupils.

The Secretary, in placing the charges before the Board, pointed out that Miss Gregory did not deny that she had signed the schedules. It would be for the Board to take into consideration the motive behind the act. The depth of guilt varied with the intent. Did Miss Gregory sign the schedules "with an innocent mind," honestly believing herself justified in so doing? Or did she so act in order to facilitate the training of more pupils than her material allowed?

The only witness called was Miss Hannam, Secretary of the London Obstetrical Society, of whom the Board asked a few questions bearing on the procedure of the Society with regard to their examinations. Miss Gregory's solicitor addressed the Board on her behalf, and she was herself examined. The Board then deliberated in private, their decision being subsequently announced on the readmission of Miss Gregory and the Press.

The Chairman, addressing Miss Gregory, said that the Board had considered all the circumstances of the case and had decided not to remove her name from the Roll, but they found her guilty of serious misconduct, and desired that she should be severely censured and cautioned. He further pointed out that if the examination for which such falsely-signed schedule had been sent in, had been that of the Central Midwives Board, instead of the Obstetrical Society of London, the penalties incurred would be of a most serious nature. The Act laid down under Sections 11 and 12 that "any woman who procures or attempts to procure a certificate under this Act by making or producing, or causing to be made or produced, any false or fraudulent declaration, certificate, or representation, either in writing or otherwise, shall be guilty of a misdemeanour, and shall on conviction thereof be liable to be imprisoned with or without hard labour, for any term not exceeding 12 months." Also that "any person wilfully making or causing to be made any falsification in any matter relating to the Roll of midwives shall be guilty of a misdemeanour, and shall be liable to be imprisoned with or without hard labour for any term not exceeding 12 months."

### THE ADJOURNED MEETING.

At the conclusion of the special meeting the Board proceeded to take adjourned business, and having decided to consider applications for approval of certificate, and for recognition as teachers and of midwives for signing forms III. and IV. in camera, the last item on the agenda was taken first. This was a resolution moved by Miss Wilson, and seconded by Dr. Cullingworth :—

That the following note be appended to Forms I., III., IV. and V. in the schedule to the rules:—

N.B.—By Section 12 of the Midwives Act, 1902, any person wilfully making, or causing to be made, any falsification in any matter relating to the roll of midwives is guilty of a misdemeanour, and is liable to severe penalties.

The resolution was passed unanimously, and the secretary was instructed, after obtaining the sanction of the Privy Council, to have the note printed in red ink at the foot of the forms in question.

The representatives of the Press were then asked to withdraw.

The following medical practitioners were approved as teachers during the sitting of the Board in camera: A. G. Everard, L.R.C.P.; Heaton C. Howard, M.R.C.S.; F. Rees, M.D.; B. A. Eaton, M.B.; E. E. Norton, M.R.C.S.; M. J. Robinson, M.D.; and G. de G. Griffith, M.R C.S.

The following certified midwives were approved for the purpose of signing Forms III. and IV.:—Edith Cotsworth, Sarah Grayson, Jane Jessie Rae, and Annie Sarah Standfield.

### Everybody's Opinion.

[Correspondence on all subjects is invited, but we cannot in any way be responsible for the opinions expressed by our correspondents. No communication can be entertained if the name and address of the correspondent are not given as a guarantee of good faith, but not necessarily for publication. All correspondents should write on one side of the paper only.]

### WOMEN INSPECTORS OF MIDWIVES.

"W." writes: I have to thank you for publishing my letter and for your reply, but at the risk of being considered very troublesome I must remind you that every properly trained midwife is expected to be able to detect any abnormal conditions which may arise during pregnancy, labour, or the puerperium. In fact, I have always understood that part of our superiority to the untrained "Gamp" consisted in our ability to do so and in our readiness to ask for medical advice. I still fail to see the superior fitness of a fully-trained nurse for the post of inspector or that of a lady doctor, unless the inspector is supposed to treat the abnormal conditions, which would obviously be very unfair to the medical men already established in the neighbourhood.

### STATE REGISTRATION.

SISTER ELLEN writes: I am interested in reading the numerous articles upon registration of nurses, and I do not think we are much nearer the solution of the difficulty. If registration becomes compulsory we should still have the inefficient nurse amongst us in the same way as we have medical men who differ vastly in their abilities. In my opinion the best plan to avoid and get rid of the half-trained nurse, would be to compel all nursing homes to be registered, and to have their list of nurses inspected by a competent authority frequently, and if found to have nurses without having proper training yet taking fees as fully trained, let the proprietor be punished as in any other trade. I believe that nursing homes are the greatest offenders as so many nurses who prove unfit for hospitals from various causes drift into such places with a partial training. To ask full fees for half-trained nurses is most unfair to the patient. Medical men could assist largely if they would refuse to employ any nurses who have not received three years' training or are not able to produce a certificate to that effect. I believe that by this means the unskilled nurse would speedily disappear and State registration would be unnecessary and undesirable.

### WOMEN ON HOSPITAL BOARDS.

"EX QUEEN'S NURSE" writes: Permit me as a constant reader of THE HOSPITAL to congratulate you on your able exposition of amateurs on hospital boards. Women in the capacity of management on any public or private committee cannot possibly help doing much more harm than good, unless they are specially gifted in mind, and suitable in purpose to meet all the technicalities of a board of management, where the interests of others are concerned, more particularly in the immense responsibilities peculiar to hospital administration. To take a local committee of any nursing association, for instance, wherever you will, as a general rule the amateur reigns supreme. It is this grievous defect which often causes an utter impossibility for a professionally-trained nurse (who is often not without some fault) to carry out freely, and in a happy spirit of conscientiousness according to her ability, her arduous duties. She is often handicapped and distressed by having to contend with so many things, both great and small, which are unnecessarily brought about in an unpleasant and undignified manner by administrative unfitness of otherwise good women, few of whom possess those qualities which, when cultivated, would entitle them to justly call themselves "not amateurs," and possessing a "thorough understanding." Had they these qualities they would command all the respect which every proper and fair-minded person would generously pay towards this much-abused yet much-loved sex. As far as my experience goes, almost every woman who happens to be appointed on a public board seems to consider that the mere fact of her having been chosen renders her a competent authority to dictate to every official under the control of this board.

MISS E. L. C. EDEN writes: I beg to thank you for your courteous and careful answer to my question as to why you think women must necessarily be useless on Hospital Boards. The explanation of my attitude is that I am thinking of provincial hospitals, not London ones. In the country a hospital board does not consist of experts. There are retired military men and country gentlemen, as well as business men on it. In such a body I hold that a capable woman with certain qualifications would, by comparison, be an expert. She would understand household economy, the details of management and the needs of women and children. I remember a member of the board at a hospital I was in, suggesting by way of economising ward fires that the kettle for fomentations, etc., should be boiled and kept in the sisters' bed-sitting room! Men have no grasp of detail as a rule. I do not maintain, as you suggest, that "any woman, because she desires to be upon a hospital committee, who has never had any special training, or who possesses no technical knowledge or experience of business would be able, if elected, to render any useful service" any more than I should maintain this of a man—but I do maintain that there are a certain number of women who have special qualifications. There are retired matrons of hospitals, women who have studied sanitation, women who have gone deeply into the science of household economy, and others who would surely work well and faithfully on country hospital boards. "Men buildyth houses; woman makyth homes." Even for the sake of this fact let us have mixed boards.

[Miss Eden, it will be seen, concedes our points. Of course there may be here and there special circumstances which can best be dealt with on their merits, if and when they arise. There can be no difference on the main issue amongst those who possess the necessary experience and knowledge. —ED. THE HOSPITAL.]

### NO NIGHT NURSE FOR NORTHALLERTON INFIRMARY.

"NURSE EVA" writes: Please do not think for one minute that the Northallerton are the only Board of Guardians who consider one nurse sufficient to attend to the needs of their workhouse patients. In small workhouses, where the number of patients are only 20, or occasionally rather under, the need of a night attendant is either unthought of or ignored. That a pauper should be cared for by night as well as by day is ridiculous, and a nurse who suggested such a thing would expect to be told that she pampers her people instead of making them glad to "get up" and "go out." Or, if a patient is taken worse in the night, perhaps becoming delirious and getting uncovered, or even out of bed, why "Nurse should be there; what else is she paid for?" The patients must be most carefully watched and tended during the 12 hours of day, and doctor's orders most carefully carried out; in fact, matron will take the trouble to put on her carpet-slippers 12 times a day (if it suits her convenience) to see that nurse is doing her duty, but . . . in the evening time "matron is tired, and, after all, the infirmary is nothing to do with her?" What about the next 12 hours? If nurse needed watching when all were awake, and the daily work with its worry and noise would keep her up to the mark, what will she do in the silent hours of night if attention be needed? However, want of thought on the part of the guardians had not occurred to me until reading the remarks on the "Northallertons" in THE HOSPITAL, but what had seemed rather hard to me was one of many similar remarks of matron's. Of course, I must admit, she is untrained. Having had several bad cases during the last few weeks and consequently made many night visits to the wards, I quite naturally began to get poorly myself, but was hoping for quieter times in which to pull up my strength. Then three nights in succession I was called up, and the fourth night disturbed after having only retired half an hour. I stayed up all night, worked on until 2 P.M. next day and then went to my room for a few minutes, tired and faint, of course, only to be found there by matron who observed that I was "too delicate and refined for the work." Can any reader kindly tell me how many hours in succession I must be able to work without showing signs of fatigue before I can be considered fit for a Poor-law nurse? Being faint-hearted I have resigned.

### Death in Our Ranks.

BY the death of Miss Alice Watson, which occurred from chronic Bright's disease on January 15th, writes a correspondent, the English colony here in Rome has sustained a real loss. "Herself the daughter of a medical man, Miss Watson chose nursing for her profession, and after training in Liverpool and doing the work of district nurse in London until her health suffered, she came to settle in this city about eleven years ago. During this time she made herself much appreciated as an efficient nurse and masseuse, and has also done a very good work for the English community by establishing and directing a small nursing home, and thus helping to provide the residents and visitors with thoroughly-trained nurses in time of sickness. Not only her professional ability, but her generous and unselfish nature, has won for her the esteem and affection of all with whom she came in contact. This was well seen by the kind care and friendship with which she was surrounded during her painful illness. Although entirely dependent on her work for her living she was always ready to help those poorer than herself, and spared herself neither time nor trouble in the discharge of her duties."

Nurse Mary Drayton died on January 9th at Nice. She was trained at the Poplar and Stepney Sick Asylum and was afterwards on the staff of the British Hospital, Algiers. She then joined the private staff of the Royal United Hospital, Bath. Subsequently she went a second time to Algiers and there contracted phthisis from a patient. She was never able to work again and grew gradually weaker. She died at the early age of thirty.

### Novelties for Nurses.

### THE PEACE PILLOW.

THE sufferer from chronic insomnia almost invariably has many advisers but only too often he learns to despair of their proposals. Should he be tempted to appeal to narcotic drugs he soon learns that these have serious risks and disadvantages. Hence the suggestion of a pillow endowed with specially soothing properties seems both ingenious and appropriate. In the "Peace Pillow" this is accomplished by impregnating a cushion possessing attractive physical qualities with agents of soporific and soothing virtues. The idea is well carried out and the pillow may usefully be remembered in cases where obstinate wakefulness is a troublesome feature.

### Appointments.

CHESHUNT ISOLATION HOSPITAL.—Miss M. A. Chapman has been appointed matron. She was trained at Fir Vale Infirmary, Sheffield. She has since been charge nurse at the Western Fever Hospital, Fulham, a member of the staff of the General Nursing Institute, Mandeville Place, W., and has been attached to a private nursing home.

CITY OF LONDON HOSPITAL FOR DISEASES OF THE CHEST.—Miss H. Smith has been appointed staff nurse. She was trained at Bethnal Green Infirmary, London, and has since been staff nurse at St. Luke's Home, Pembridge Square, London.

CORNWALL COUNTY COUNCIL LECTURER.—Miss Minnie Leng has been appointed lecturer in the St. Ives district She was trained at Guy's Hospital, London, and holds the Guy's medal for five years' service, and the South African war medal. She has been matron of the Wolverhampton and Staffordshire Hospital.

COTTAGE HOSPITAL, SOUTHWOLD, SUFFOLK.—Miss Ellen Supple has been appointed staff nurse. She was trained at the Meath Hospital, Dublin.

DEWSBURY AND DISTRICT GENERAL INFIRMARY.--Miss Helen Edgar has been appointed charge nurse. She was trained at the Royal Infirmary, Manchester.

FARNHAM UNION INFIRMARY.—Miss Clara A. Tisdell has been appointed superintendent nurse. She was trained at Mile End Infirmary, London, and has since been staff nurse and night superintendent at the same institution, superintendent nurse at Whitechapel Union Infirmary and at the Tonbridge Union Infirmary. She holds the L.O.S. and Central Midwives Board certificates.

VICTORIA HOSPITAL, FOLKESTONE.—Miss Curtis has been appointed sister and Miss Wylie night sister. Miss Curtis was trained at the Metropolitan Hospital, London, and Miss Wylie at King's College Hospital, London.

WATFORD ISOLATION HOSPITAL.—Miss Irene Bathie has been appointed sister. She was trained at the Sanatorium, Huddersfield, and Bethnal Green Infirmary, London, and has since been sister at the Sanatorium, Huddersfield.

### TRAVEL NOTES AND QUERIES.

BY OUR TRAVEL CORRESPONDENT.

ROYAU-LES-BAINS (S M.S.).—It is considered healthy and is rather in favour as a winter residence. I cannot tell you the price of a furnished villa, and my feeling is always against renting one for a short period. If you wish to do so, however, the matter is simple enough; go to Hôtel des Voyageurs or Hôtel du Commerce for a few days and make personal inspection of the villas, a list of which will be given you by a house agent. Your hotel proprietor will tell you to whom to apply. I should not think of a villa for such a limited period; the expenses run up so alarmingly and there is no time to achieve a balance satisfactorily, as might be the case during a residence of six months. If you like a sea voyage you might go to Bordeaux by steamer. If I can help you further let me know.

THE CHANNEL ISLANDS (A. B.).—There is very little difference between the Weymouth and Southampton lines. The former is rather shorter. You can see all the islands fairly well in three weeks, and the £15 each is amply sufficient, indeed, I think it may be done comfortably for £12 10s. I think your best plan would be to spend one week in Jersey, one in Guernsey, and one in Sark. If you do not like quiet, you would not care for a week in Sark, and had better only remain there two days. It is to my mind very charming and not so full of tourists as the other islands. The hotel you mention in Guernsey is still there—terms 7s. 6d. per day. There are others cheaper than this if you wish to do the thing economically; good accommodation can be had both in Jersey and Guernsey for 5s. 6d. per day. Sark is dearer, because it is quieter and there is less competition. Yes, you can visit the smaller islands from Jersey and Guernsey. Do not waste time over Herm. La Coupée is in Sark, a wonderfully raised road like the back of a razor; it connects Great and Little Sark and is worth a visit to the island, were there nothing else to see. A useful guide book for you is Black's Channel Islands, price 2s. 6d. You have time before you, so that you can thoroughly study the subject. Write me again if you want further help. June would be a nice month for your holiday if that suits you. In July and August prices rise so much.

### Echoes from the Outside World.

### Movements of Royalty.

THE KING AND QUEEN arrived at Windsor on Saturday. On Sunday they commemorated the fourth anniversary of the death of Queen Victoria by attending a memorial service at Frogmore Mausoleum. The Prince and Princess of Wales and other members of the Royal Family were present. The Archbishop of Canterbury conducted the impressive service, which concluded with a special prayer for the Royal Family and the Benediction. Between two and three thousand persons were admitted to the Mausoleum during the afternoon, and a number of wreaths were laid round Queen Victoria's tomb.

### Accession Day.

THE fourth anniversary of the King's Accession was observed on Sunday in many of the churches. At St. Saviour's, Maida Vale, the Bishop of London, preaching on the words, "Fear God, honour the King," said, in the course of his sermon, "that the cool courage which King Edward VII. displayed in the face of imminent death, his countless acts of kindness to the poor, his steady support of hospitals and other philanthropic institutions in our midst, and his tact and wisdom in dealing with foreign countries, had won him the ever-increasing esteem and love of his people. Therefore, from their hearts they prayed that day, "God Save the King." The anniversary was observed at Windsor on Monday, a Royal salute being fired in the Long Walk in celebration. In London a Royal salute of 41 guns was fired in St. James's Park at noon. Flags were flying on most of the public buildings.

### The Shadow of Revolution in Russia.

INTEREST in Russia centres this week not in the war with Japan, but in the startling events which took place on Sunday in the streets of St. Petersburg. A number of the workmen on strike carried out their declared intention of repairing to the Winter Palace in order to present a petition to the Tsar, but found all the streets strongly held by troops, who kept them back. The Palace itself was garrisoned by Cossacks. Collisions soon took place, and though in at least one instance the infantry laid down their rifles and refused to fire, the Cossacks and Uhlans charged the crowds without hesitation. The people in several cases offered a stubborn resistance, and large numbers, including women and children, were killed and wounded. A determined attempt was made to rush the Palace Square, but it was defeated by the troops, who eventually drove back and dispersed the crowd. Father Gapon, moving at the head of the demonstration with cross upraised, was shot in the arm, and his *confrère*, Father Sergius, was killed. There was no renewal of disturbances in the central parts of St. Petersburg on Monday, but the ferment continued in the outlying industrial quarters. An official narrative of the origin of the strike ascribes it largely to political agitation, to revolutionary designs, and to "the fanatical utterances of Father Gapon." It also describes the action of the troops in repressing the disturbances, and states that up to eight o'clock on Sunday morning the number of persons killed was 96 and of wounded 333. This is entirely at variance with independent accounts which give the number of killed as far higher, and of wounded at upwards of 2,000. On Tuesday there was a lull in the capital, but the strike movement is spreading in Moscow and other towns. The Tzar has issued a decree appointing General Trepoff, who was recently shot at in Moscow, military governor of St. Petersburg, and conferring upon him drastic powers.

### Terrible Railway Accident in Yorkshire.

EARLY on Thursday morning last week a terrible accident occurred on the Midland Railway at Storr's Mill, near Cudworth Junction, between Barnsley and Leeds. The Scotch up express, while running at a speed of about 50 miles an hour, dashed into a mail train from Leeds to Sheffield, which was travelling slowly. The force of the impact was tremendous, and both trains were wrecked and caught fire. Six persons were killed on the spot, and two of the injured, who numbered 15, have since expired. Bad as it was, the disaster would have been far worse if the guard of a goods train on an adjoining line had not shown great presence of mind. Knowing that a down express from London to Scotland was due a few minutes after the collision he managed to give the alarm and the train was pulled up, merely grazing the wreckage with which the permanent way was completely blocked. The killed included two school boys on their way to Bradfield College, and Mr. Robert Brough, a well-known artist, died in the Royal Hospital, Sheffield, after much suffering, on Saturday evening.

### Colliery Explosion in Wales.

A DISASTROUS explosion occurred in the Elba Pit, Gowerton, near Swansea, on Saturday morning, causing the death of 10 men and injuring several others. For some years the colliery has been working the 3 feet and 5 feet seams, but during the past two years a 6 feet seam has been reached by a low-measure drift, which extends nearly 700 yards. A collier who was engaged at the time at the very extreme end of the level has stated that "he lost himself for many minutes." He heard people crying and praying round him, and on looking round for his mate, who had momentarily left him to go for a tool, he found that the latter was amongst the killed.

### The State Maintenance of Children.

A CONFERENCE of delegates from British Labour organisations, Socialist and other bodies, was held in the Guildhall last week, on the question of the State Maintenance of Children. Sir John Gorst, M.P., who presided, said that he was only in favour of the State maintenance of children in need, but a resolution in favour of the cost of the maintenance of children being borne by the National Exchequer, "As a necessary corollary of universal compulsory education, and as a means of arresting the physical deterioration of the industrial population" was carried by an overwhelming majority. The Government, as "a step towards State maintenance," were called upon to introduce "such legislative measures as would enable local authorities to provide meals for children attending common schools."

### Death of Mr. G. H. Boughton, R.A.

THE death of Mr. G. H. Boughton, R.A., occurred suddenly on Thursday last week. Mr. Boughton complained of fatigue on the previous Saturday, but he seemed to have recovered; and his friends were greatly shocked to hear that he had been found dead in the studio of his beautiful house at Campden Hill from heart disease. The deceased artist was born at Norwich in 1836, but spent his early years in America. In 1860 he went by the aid of students to Paris to study, and formed the acquaintance of Whistler and others. Two years later he came to England, and was so well received that he decided to remain here. He exhibited his first picture at Burlington House in 1863. In 1879 he was elected an associate of the Academy, and in 1896 a full academician. His special subjects were the "New England of the days of Evangeline" and "Hester Prynne," and the frozen canals and quaint costumes of north Holland. Mr. Boughton was also a fine landscape painter.

### Notes and Queries.

**REGULATIONS.**

**The Editor is always willing to answer in this column, without any fee, all reasonable questions, as soon as possible.**

**But the following rules must be carefully observed :—**

**1. Every communication must be accompanied by the name and address of the writer.**

**2. The question must always bear upon nursing, directly or indirectly.**

**If an answer is required by letter a fee of half-a-crown must be enclosed with the note containing the inquiry.**

*Sanatorium.*

(124) Can you tell me of a sanatorium for the open-air treatment of consumption where a girl of 21 can be taken? Family not able to pay anything, but small sum might be subscribed by clergyman and others.—*G. W.*

See the list of sanatoria, published by the National Association for the Prevention of Tuberculosis, obtainable from the Scientific Press, price 7d. with postage.

*Victoria Order of Nurses*

(127) Will you kindly tell me if there are any training homes in connection with the Victoria Order of Nurses in England?—*B. W. S. C. H. N.*

If you mean Queen Victoria's Jubilee Institute for Nurses, there are many homes and institutes connected with it. Write to the General Superintendent, 120 Victoria Street, Westminster, S.W.

*The Treatment of Rheumatism by Salacin.*

(128) Can you tell me where I can obtain a treatise on the treatment of rheumatism by salacin?—*L. M.*

Any good medical text-book will give an account of the treatment.

*District Midwife.*

(129) Will you kindly tell me if there is any place in Liverpool I can apply to to get an appointment as district midwife?—*W.*

Write to the Town Clerk of Liverpool. The Midwives' Institute might also help you. Write to the Secretary, 12 Buckingham Street, Strand, W.C. If you have not yet been registered you should register at once. "How to Become a Midwive." published by the Scientific Press, post free 1s. 1d., might be of help to you.

*Indian Marines.*

(130) Can you give me particulars of how to join the Indian Marines as male nurse or dispenser?—*Ceylon.*

Write to the Director of Transports, the Admiralty, S.W.

*Burial of Still-born Infant.*

(131) Information required as to length of legal notice to incumbent of parish for interment of still-born infant.—*Veritas.*

There is no legal length of notice to be given to the incumbent, but a still-born child cannot be buried unless the person in control of the burial ground is furnished with (*a*) the certificate of a registered medical practitioner who was present at the birth, or examined the body, that the child was not born alive; (*b*) a declaration to the like effect signed by some person who would, if the child had been born alive, have been required to register the birth, and stating that no registered medical practitioner was present at the birth, or that his certificate cannot be obtained; or (*c*) if there has been an inquest, an order of the coroner.

*Parish Nurse.*

(132) I should be much obliged if you would send me particulars of the parish you mention in London requiring a nurse where a salary cannot be guaranteed. I should like to know if there would be plenty of nursing?—*L. J. A.*

We have no particulars of the parish, but if you like to send a letter to "*Z. S.*, query No. 104" it will be forwarded to the lady who made the inquiry.

*Midwives.*

(133) Can you tell me of an association that a certificated midwife, L.O.S., could join? I do not mean to live in, but to pay commission on cases.—*Anxious.*

Write to the Midwives' Institute, 12 Buckingham Street, Strand, W.C.

**Handbooks for Nurses.**

| | Post Free. |
|---|---|
| How to Become a Nurse: "The Nursing Profession" ... | 2s. 4d. |
| "Nurses' Pronouncing Dictionary." Terms and Treatment... ... ... ... ... ... ... ... | 2s. 0d. |
| "A Handbook for Nurses." Dr J. K. Watson ... ... | 5s. 4d. |
| "Elementary Physiology for Nurses" ... ... ... | 2s. 0d. |
| "Swedish Massage and Movements" ... ... ... | 2s. 6d. |

Of all booksellers or of The Scientific Press, Limited, 28 & 29 Southampton Street, Strand, London, W.C.

### For Reading to the Sick.

### THE SIGNIFICANCE OF DEATH.

THE graves grow thicker, and life's ways more bare
  As years on years go by;
Nay! Thou hast more green gardens in Thy care,
  And more stars in Thy sky!

Behind, hopes turned to griefs, and joys to memories,
  Are fading out of sight:
Before, pains changed to peace, and dreams to certainties,
  Are glowing in God's Light.

*Lyra Mystica.*

"What is the significance of Death?"

Clearly Death is a fact; a fact of intimate and universal interest; it is true of us all, we must die.

What is the significance of Death? Death in Christ is an accident in immortality. The great Unity of Life lasts on. The immortal life knows no break in its continuity, only here it is a life sin-stained, sorrow-laden; there, sin is gone and sorrow ended, when "in Christ" the living spirit passes the gates of the grave.

And further, one of the bitterest pangs of life is the pang of the parting of friends. Now, death "in Christ" is the entrance to a land where partings are no more.

It is rest from labour, it is the close of struggle, it is the sleep of the blessed, it is the fulfilment of the earthly pilgrimage; it is indeed solemn, for it is the opening of an eternal future, but it is the passage to the Audience Chamber, it is admission to the unimagined blessedness of the Presence of Christ. . . .

To live in Faith is to prepare to die. Christ by His death has given us a ground of confidence in His unflagging tenderness, and it is devotion to a person, it is faith in Jesus Christ which, as it conquers the world, so it subdues the grave. . . .

Yes, it is sad to die. But resignation to the great and loving will of God, and faith in His promise and His power, if they do not destroy the sadness which must accompany our human life, which must stand with us by the grave, at least they prepare us to bear the trial with unflinching courage and supporting hope. . . .

To live in a manful and abiding sorrow for sin; to aspire with increasing efforts towards our great Ideal; to grow in the self-sacrificing love which makes the life of each a rich inheritance for all; to deepen in a steadfast trust in the Father, Who is revealed to us in the tender love of the Divine and Human Son; this is to rob death of its terrors, this is to tread the rough and splendid path of the Passion. This is to enter into the meaning of that great assurance—"O death of Christ, the death of death to me!"

*Canon Knox Little.*

Subtlest thought shall fail, and learning falter,
  Churches change, forms perish, systems go,
But our human needs, they will not alter,
  Christ no after-age shall e'er outgrow.

Yea, Amen! O changeless One, Thou only
  Art life's guide and spiritual goal,
Thou the Light across the dark vale lonely—
  Thou the eternal haven of the soul!

*J. C. Shairp.*

